# Higher Content but No Specific Activity in Gelatinase B (MMP-9) Compared with Gelatinase A (MMP-2) in Human Renal Carcinoma

**DOI:** 10.3390/cancers15225475

**Published:** 2023-11-20

**Authors:** Grzegorz Młynarczyk, Monika Gudowska-Sawczuk, Barbara Mroczko, Marta Bruczko-Goralewska, Lech Romanowicz, Anna Tokarzewicz

**Affiliations:** 1 Department of Urology, Medical University of Białystok, 15-089 Białystok, Poland; 2 Department of Biochemical Diagnostics, Medical University of Białystok, 15-269 Białystok, Poland; monika.gudowska-sawczuk@umb.edu.pl (M.G.-S.); mroczko@umb.edu.pl (B.M.); 3 Department of Neurodegeneration Diagnostics, Medical University of Bialystok, 15-269 Białystok, Poland; 4 Department of Medical Biochemistry, Medical University of Białystok, 15-089 Białystok, Poland; marta.bruczko-goralewska@umb.edu.pl (M.B.-G.); lech.romanowicz@umb.edu.pl (L.R.); anna.tokarzewicz@umb.edu.pl (A.T.)

**Keywords:** renal carcinoma, gelatinase, MMP-2, MMP-9, cancer, biomarker

## Abstract

**Simple Summary:**

Gelatinases are one of the major groups of matrix metalloproteinases. Gelatinase A which known as matrix metalloproteinase-2 and gelatinase B referred to as matrix metalloproteinase -9 play a crucial role in breaking down gelatin and various components of the extracellular matrix, including denatured collagen. It has been observed that, in cancer gelatinases are often associated with an-giogenesis, tumor invasion and metastasis.Matrix metalloproteinases have been studied for their involvement in the progression of various tumors in recent years. This paper analyses the contribu-tion of gelatinases A and B in human renal carcinoma pathogenesis, showing their opposite action both at the stage of extracellular matrix remodeling and at different stages of renal tumor devel-opment. Based on the obtained results, MMP-9 showed higher content and lower specific activity compared with MMP-2.

**Abstract:**

Gelatinases belong to a group of enzymes known as matrix metalloproteinases (MMPs). Gelatinases A and B (MMP-2 and MMP-9, respectively) are the enzymes with the highest ability to destroy collagen, primarily type IV collagen, which is an essential component of the base membrane. Hence, it can be assumed that they are involved, among other things, with the metastasis process of cancer. As a result, the objective of this study was to assess the presence, activity, and expression of selected gelatinases in human renal cancer. Healthy (*n* = 20) and clear-cell kidney cancer tissue samples (G2 *n* = 10, G3 *n* = 10) were analyzed. The presence and content of MMPs were measured using the Western blot and ELISA methods, respectively. The activity (actual and specific) was analyzed with a fluorimetric method. The presence of both investigated enzymes was demonstrated in the representative zymogram. MMP-9 showed the most intensive saturation. It has been observed that both gelatinases occur primarily in high molecular complexes in the human kidney, regardless of whether it is a control or tumor tissue. Both gelatinases were present in comparable amounts in healthy tissues of the kidney. MMP-9 showed a higher content than MMP-2 in both renal cancer grades, but we observed the enhanced activity of both gelatinases with an increase in the grade of renal cancer. A higher MMP-9 content and, on the other hand, lower specific activity in the cancer tissue suggest that MMP-9 is predominantly present in an inactive form in renal cancer. The higher activity of MMP-9 demonstrated using the zymography method may be a cause of different values of activity that depend on the phase of the carcinogenic process. The present study revealed changes in the tested gelatinases in healthy and cancerous tissues of renal cell carcinoma. Therefore, it can be concluded that matrix metalloproteinases 2 and 9 are enzymes directly involved in carcinogenesis, and hence, it seems that MMPs may have potential in the diagnosis and treatment of renal carcinoma.

## 1. Introduction

As parenchymal and paired organs, kidneys are localized in the retroperitoneal space of the human abdomen. Their main functions include the excretion of metabolism waste products, the regulation of constituents, the volume and pH of the body fluids, and endocrine and metabolic functions [1,2]. In addition, their functioning depends on the right structure of the extracellular matrix (ECM). The ECM is a highly charged, dynamic structure that serves as a support system for living cells and a key player in communication between cells [3]. Collagens, the primary extracellular proteins; glycoproteins; and elastin molecules make up this structure, which interacts with surrounding cells and forms a complex network [4,5,6]. The ECM is found in the glomeruli, tubulointerstitium, and blood vessels of the renal cortex, which are anatomically distinct locations with different functions based on their molecular constituents. Matrix metalloproteinases (MMPs) make up the majority of the enzymes involved in preserving ECM homeostasis [7,8,9,10,11].

Gelatinases are one of the major groups of MMPs that mainly degrade broken-down and denatured collagen. Gelatinase A, also referred to as neutrophil gelatinase or MMP-2, is involved in a variety of processes, including inflammation, vascular remodeling, angiogenesis, tissue healing, and tumor invasion. Gelatinase B (MMP-9) is an enzyme whose expression is induced during the intensified remodeling of the extracellular matrix in various pathological conditions [12,13,14,15]. Both gelatinases degrade different pre-digested types of collagen, where collagen type IV—a basic component of basement membranes—appears to be the most important.

Importantly, an increase in the expression of tumor cell gelatinases in renal carcinoma patients has been observed [16,17]. According to Kallakury et al. [18], there is a connection between elevated MMP-2 and MMP-9 expression and disease development with a poor outcome. The increased expression of the investigated MMPs and a higher stage in the TNM—Tumor Nodes Metastases staging system [19]—has been revealed. However, the expression of gelatinases and the level of tumor malignancy have not been found to be significantly correlated by other studies [20,21,22]. Hence, it seems that the role of gelatinases in the development of renal carcinoma is still not fully understood. The expression, content, and activity of MMP-2 and MMP-9 in malignant tissue, as compared with healthy tissue of the human kidney, were therefore assessed in the current research.

## 2. Materials and Methods

The investigation protocol was approved by the Committee for Ethics and Supervision on Human Research of the Medical University of Bialystok. All of the patients signed informed consent forms to be included in the study. 

### 2.1. Tissue Sample

The studied material comprised clear-cell kidney cancer tissue harvested from patients with cancer at the G2 and G3 stages according to World Health Organization (WHO)/International Society of Urological Pathology (ISUP) grading. To obtain as homogeneous a group of patients as possible, quite restrictive exclusion criteria were used. Patients with concomitant chronic diseases were not qualified for the study. Moreover, any patient taking medications regularly was disqualified from the above research project. The control sample was a section of the same kidney but maximally distant from the visible tumor. The control samples were evaluated histopathologically and showed no tumor lesions. 

The samples were collected from 20 patients suffering from renal carcinoma and divided into two subgroups: G2 (*n* = 10) and G3 (*n* = 10). All cases of suspected human renal cancer were referred for ultrasound examination (USG) and then confirmed via computed tomography (CT). The exception was 4 patients from the G3 group, who underwent immediate CT because of the occurrence of symptoms (hematuria in all and additional flank pain in one patient). The detailed clinicopathological characteristics of patients are presented in Table 1. The average tumor size in the Grade 2 group was 5.30 cm, whereas in the Grade 3 group, it was 7.15 cm. The mean BMI was higher in the G3 group (28.1) than in patients with G2 renal cell carcinoma (25.7). On the day of admission to the hospital, the results of basic laboratory tests were within reference ranges. Moreover, no distant metastases were found in any patient from the entire study group.

Immediately after excision, the tissues were rinsed with 0.9% NaCl and stored at −70 °C. The material was collected during surgical procedures (open nephrectomy) performed at the Clinic of Urology of the Medical University of Bialystok.

Tissue samples for biochemical analysis were prepared according to the procedure described in our previous publications [23,24].

### 2.2. Gelatin Zymography

The zymography technique was used to detect the presence of MMP-2 and MMP-9 in the studied material [25]. The tissue extracts were applied to 1% SDS/10% polyacrylamide gel, and electrophoresis was run at a constant voltage (150 V). After electrophoresis, SDS was removed via incubation in 2% Triton X-100 at 37 °C for 30 min. The gel was then transferred to 0.05 M Tris-HCl buffer (pH 8.0) containing 5 mM CaCl_2_, incubated at 37 °C for 18 h, and stained with 1% Coomassie Brilliant Blue R-250. The gelatinolytic activity was detected as clear bands against a blue background of undegraded substrate. 

### 2.3. Total Content Evaluation with ELISA 

The content of both examined gelatinases was determined with the use of quantitative assay, the human MMP-2 ELISA Kit for Cell and Tissue Lysates provided by Sigma, and the ELISA Kit for Matrix Metalloproteinase 9 (MMP-9) provided by Cloud-Clone Corp (Cloud-Clone Corp, Katy, TX, USA). The procedure was performed according to instructions given by the manufacturer.

### 2.4. Western Blot

Then, 20 μg samples of protein for MMP-2 and MMP-9 were electrophoresed on 10% SDS-polyacrylamide gel [26]. After electrophoresis, proteins were transferred onto nitrocellulose membranes (Sigma-Aldrich; Saint Louis, MO, USA) at 100 mA for 1 h. Non-specific binding sites were blocked with 5% non-fat milk in TBS-T (20 mM Tris-HCl buffer, pH 7.4; 150 mM NaCl; 0.05% Tween-20) for 1 h. Afterward, they were incubated with anti-human MMP-2 (R&D systems, Minneapolis, MN, USA) or MMP-9 (R&D Systems) antibody solution overnight at 4 °C and subsequently washed in TBS-T. Membranes were then exposed to the secondary antibody conjugated to alkaline phosphatase for 1 h at room temperature. The bands were visualized using BCIP/NBT reagent (Sigma). The molecular mass of the MMPs was estimated according to the molecular mass markers (BioRad, Hercules, CA, USA). Finally, representative results were presented.

### 2.5. Gelatinase Activity

An activity assay was performed using the fluorimetric method, which was described previously [23,24,27,28]. Gelatinases were first isolated on a black 96-flat-bottom-well microplate (Greiner Bio-One, Rainbach im Mühlkreis, Austria) that was precoated with specific anti-human MMP-2 or MMP-9 antibodies (R&D Systems, the same as was used in the Western blot analysis). The extract samples (100 μL) were added to the wells for the immobilization of MMP-2 or MMP-9, and the microplate was incubated overnight at 4 °C. After enzyme immobilization, the remaining proteins were washed out with TBS-T buffer (50 mM Tris/HCl pH 7.4, 0.9% NaCl, 0.05% Tween 20). To measure MMP activity, 100 μL of 50 mM Tris/HCl buffer, pH 7.5, containing 10 mM CaCl_2_, 150 mM NaCl, and 0.025% Brij 35 with MCA-Pro-Leu-Ala-Cys(p-OMeBz)-Trp-Ala-Arg(Dpa)-H2 (Merck, Darmstadt, Germany) as a fluorogenic substrate (4 μM final concentration) was used. The microplate was incubated at 37 °C for 60 min with gentle shaking. The enzymatic reaction was stopped by the addition of 25 μL of 100 mM EDTANa_2_. Degradation of the fluorogenic substrate via MMP was measured with a multimode microplate reader (Tecan Infinite® 200 PRO, Männedorf, Switzerland) with excitation and emission wavelengths set at 325 nm and 393 nm, respectively. The amount of decomposed substrate was quantified based on the calibration curve, which was prepared for 7-amino-4-methylcoumarin (Sigma) as a standard. The activity of the tested enzymes was presented as actual activity and specific activity. Actual activity was calculated per total protein content. Specific activity was calculated based on the content of a specific MMP measured with the ELISA method. 

### 2.6. Protein Determination

The protein amount was determined using the Bradford method [29]. 

### 2.7. Statistical Analysis

All content and activity results for both MMP-2 and MMP-9 were checked for normality using the Shapiro–Wilk test. The results of this test confirmed the normal distribution of most of our measurements. Student’s *t*-test was used to determine statistical significance results with the normal distribution. The Mann–Whitney U test was used for results with a non-parametric distribution. A value of *p* < 0.05 indicated that the difference between the compared groups of results was statistically significant. All results of our measurements and statistical analyses are presented in supplementary materials (Table S1). 

The results for the MMP content and activity are presented as the mean value and standard deviation (SD) obtained for 10 tested samples of each group. The MMP content is presented in nmol/g of protein, while activity is presented in microkat/kg of protein. 

## 3. Results

### 3.1. Zymography

**Figure 1:**
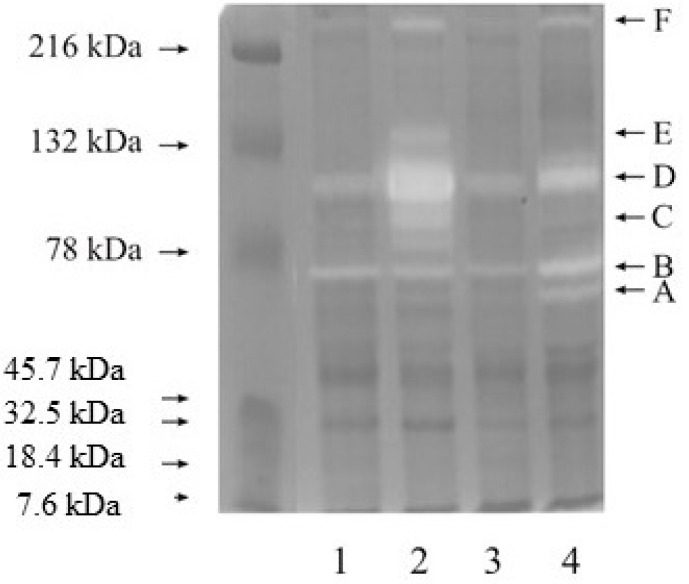
Zymography of control samples and kidney cancer (lane 2—G2 grade, lane 4—G3 grade). Each sample was applied in a quantity equal to 20 µg of protein.

The zymography analysis is presented in Figure 1. Bright bands marked with letters A–F indicate the sites of gelatin degradation, that is, the sites of metalloproteinase activity. A weakly saturated band, A, represents the molecular weight of 62 kDa, which matches the mass of an active form of MMP-2. It is present in every investigated tissue. The band marked with the letter B is more intensive and discolored. It represents 72 kDa, which is the mass of the latent form of MMP-2. The highest activity of both forms of MMP-2 was observed for G3 kidney cancer. Active (86 kDa) and latent (92 kDa) forms of MMP-9 correspond to band C and band D, respectively. The highest degree of gelatin degradation caused by both forms of MMP-9 was observed for G2 kidney cancer. Bands E and F may correspond to high molecular complexes in the enzymes (Figure 1).

### 3.2. MMP-2 and MMP-9 Content 

**Figure 2:**
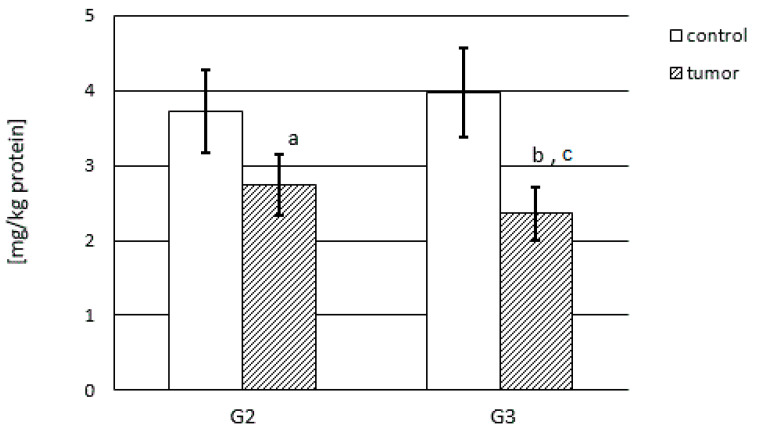
MMP-2 content in human kidney cancer at different stages and in control samples: a—*p* < 0.01; b—*p* < 0.001, cancer in relation to control; c—*p* < 0.05, G3 stage in relation to G2 of kidney cancer.

**Figure 3:**
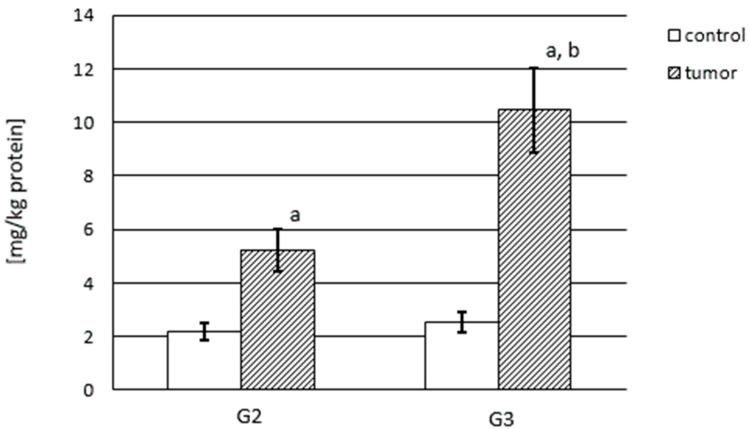
Content of MMP-9 in human kidney cancer at different stages and in control samples. a—cancer in relation to control; b—G3 stage in relation to G2.

The MMP content was calculated per kg of total protein content and was considerably different for the control samples compared with the renal cancer tissue and is shown in Figure 2 for MMP-2 and in Figure 3 for MMP-9. 

MMP-2 was present in a higher amount in control samples as compared with cancerous samples (*p* < 0.001). The G2 and G3 tumor samples showed substantially lower amounts of MMP-2. Moreover, MMP-2 was present in lower amounts in the G3 stage in relation to G2 kidney cancer (*p* < 0.05). The G2 tumor contained almost 25% less enzyme, while the G3 tumor showed as much as 40% less enzyme content in comparison with the respective control samples. Thus, progressing tumor invasiveness is characterized by a small drop in MMP-2 concentration (Figure 2).

The control samples showed only slightly less MMP-9 content than MMP-2, i.e., about 2 mg per kg of total protein. The MMP-9 content in kidney cancer was significantly higher in relation to the control samples (*p* < 0.001), and it was 136% in the G2 stage and 318% in the G3 stage. The enhanced MMP-9 content significantly progressed with the developing tumor invasiveness. There was a significant difference in the MMP-9 content in the G3 stage in relation to G2 kidney cancer (*p* < 0.001) (Figure 3).

### 3.3. Western Blot Analysis of Investigated Gelatinases

We used relevant specific primary antibodies to evaluate the expression of both gelatinases. In accordance with the best resolution of electrophoretic separation and the next Western blot picture, the same quantity of protein was applied to the gel for each MMP.

#### 3.3.1. Expression of MMP-2

**Figure 4:**
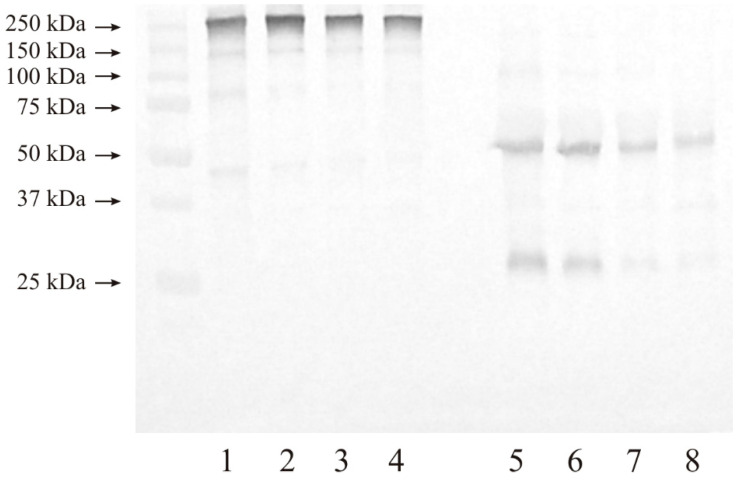
MMP-2 Western blot analysis of the control samples and both grades of kidney cancer. The same amount of protein (20 µg) was applied in each lane. Electrophoresis was run in non-reducing conditions (lanes 1–4) and reducing conditions (lanes 5–8). Lanes 1, 5—control samples for G2 kidney cancer; lanes 2, 6—G2 stage of kidney cancer; lanes 3, 7—control samples for G3 kidney cancer; and lanes 4, 8—G3 stage of kidney cancer.

A representative blot for the presence of MMP-2 in the control samples and renal tumor tissues is shown in Figure 4. In non-reducing conditions, all tested samples demonstrated the presence of an intensive band at 250 kDa, which may indicate the presence of high molecular complexes of MMP-2 (Figure 4, lanes 1–4). Additionally, both control and kidney cancer samples showed bands at 150, 85, and 45 kDa. No other bands with lower masses in non-reducing conditions were visible.

In reducing conditions, none of the samples tested (Figure 4, lanes 5–8) showed the presence of macromolecular complexes that were present under non-reducing conditions. Additionally, all tested samples demonstrated the presence of bands at 50 and 28 kDa (Figure 4, lanes 5–8).

#### 3.3.2. Expression of MMP-9

**Figure 5:**
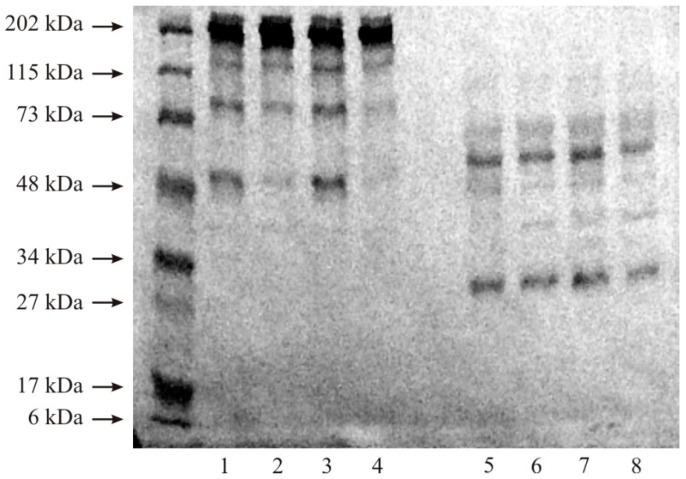
MMP-9 Western blot analysis of the control samples and both grades of kidney cancer. The same amount of protein (20 µg) was applied in each lane. Electrophoresis was run in non-reducing conditions (lanes 1–4) and reducing conditions (lanes 5–8). Lanes 1, 5—control samples for G2 kidney cancer; lanes 2, 6—G2 stage of kidney cancer; lanes 3, 7—control samples for G3 kidney cancer; and lanes 4, 8—G3 stage of kidney cancer.

Figure 5 demonstrates a Western blot analysis of MMP-9. Separation in non-reducing conditions provided bands at 115, 73, and 48 kDa in all studied samples (Figure 5, lanes 1–4). The presence of dark intensive bands of more than 115 kDa may suggest the presence of MMP-9 in macromolecular complexes. 

Electrophoretic separation in the presence of disulfide bridge reducing agents (Figure 5, lanes 5–8) resulted in the appearance of only two distinct bands at 55 and 30 kDa. These bands were present in both control samples and in both stages of kidney cancer. 

### 3.4. Activity of Gelatinase A and B

The activity of the enzymes tested is presented as actual activity and actual specific activity. Actual activity is expressed per total protein content. Based on the gelatinase content, measured with the ELISA method, we calculated the specific activity of the MMPs. The specific activity we express per kg of the appropriate enzyme protein. 

#### 3.4.1. Actual and Specific Activity of MMP-2

**Figure 6:**
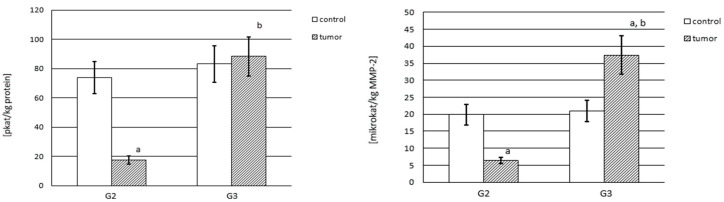
MMP-2 actual and actual specific activity in control samples and both grades of kidney cancer. a—cancer in relation to control. b—G3 stage in relation to G2 kidney cancer.

Figure 6 shows the actual and actual specific activity of MMP-2. The actual activity of MMP-2 is expressed in pikokatals per kg of protein content. It was found that in both control samples and G3 tumor tissue, actual activity was similar. Furthermore, only the G2 tumor showed very low MMP-2 actual activity, about 23% of the activity of the respective control samples (*p* < 0.001). An increase in kidney cancer grade also entailed a considerable increase in the measured activity. There was a significant difference between G3 and G2 (*p* < 0.001). The MMP-2 activities in both grades of cancer tissue significantly differed. The actual activity of MMP-2 measured in the G3 tumor was over five times higher compared with the G2 tumor. The results for the actual specific activity of MMP-2 are expressed in mikrokatals per kg of enzyme content. Similar actual specific activity, about 20 microkatals per kg of MMP-2, was found in both control samples. The activity of MMP-2 in G2 tumor tissue was significantly lower, almost four times lower in relation to the control samples (*p* < 0.001). However, the specific activity of MMP-2 in the G3 tumor tissue was extremely high in comparison with the G2 stage of kidney cancer (*p* < 0.001) (Figure 6).

#### 3.4.2. Actual and Specific Activity of MMP-9

**Figure 7:**
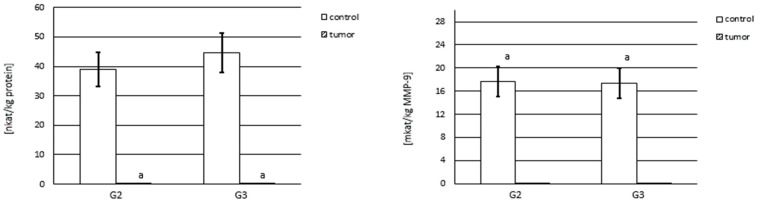
MMP-9 actual and actual specific activity in control samples and both grades of kidney cancer. a—cancer in relation to control.

Figure 7 shows the actual and actual specific activity of MMP-9 in all studied samples. MMP-9 actual activity is expressed in nanokatals per kg of total protein. Kidney tumors (G2 and G3) are characterized by a significant decrease in MMP-9 activity in comparison with the control sample (*p* < 0.001). Kidney cancer at the G3 stage showed an approximately fivefold increase in measured activity relative to the G2 stage. However, such a rise was not sufficient to reach the control sample value; the activity of the G3 tumor was still considerably lower. The actual specific activity of MMP-9 is expressed in mikrokatals per kg of enzyme content. Specific activity significantly dropped in both grades of kidney tumors relative to the corresponding control samples (*p* < 0.001). We observed over 2000 times higher activity in the control samples as compared with the G2 tumor tissue and more than 900 times higher in the control samples compared with the G3 tumor grade. 

## 4. Discussion

The human kidney contains a small amount of extracellular matrix. In addition to collagen, there are many other equally important components of the ECM: proteoglycans, structural glycoproteins, and elastin. A special layer separating epithelial tissue from connective tissue, the basement membrane, is a special layer of the ECM. The basement membrane is mainly composed of glycoproteins (laminin and nidogen–collagen) and collagen IV. Their turnover depends not only on synthesis but also on their degradation. The specificity of human renal cell carcinoma is still the subject of much research. Therefore, we decided to evaluate the content, expression, and activity of enzymes involved in the degradation of ECM components specifically in kidney tissues.

Gelatinases primarily degrade broken-down and denatured collagen. They achieve their greatest ability when degrading type IV collagen [30,31,32]. We compared the results of measurements for MMP-2 with those for MMP-9 carried out for human kidney cancer and with those obtained for control samples. The latter was used as a control sample, as it was not possible to collect kidney parts from healthy donors for ethical reasons. The postmortem collection of a human kidney results in a significant change in protein content. Such collection is not possible after several hours. By that time, the process of tissue degradation is significantly advanced. In enzymes that degrade the extracellular matrix, all components are active [33]. In freshly collected tissue taken from a living organism, the protein content is much higher than it is in tissue taken postmortem.

First, to confirm the presence of gelatinases, the zymography method was performed. With the use of gelatin as a substrate, MMP-2 and MMP-9 presence and activity were confirmed in all investigated tissues. The most intense saturation and activity were also demonstrated by MMP-9, especially in the G2 grade.

We measured the total MMP-2 and MMP-9 content in kidney extracts using the ELISA test. The content of the examined gelatinases was small but still significantly differentiated and dependent on the respective grade of cancer progression. We observed that the control samples contained similar amounts of both investigated gelatinases. This demonstrates that both enzymes may be identically important in extracellular matrix reconstruction occurring in healthy kidneys. A significant decrease in MMP-2 in both grades of cancer in contrast to a distinctive increase in MMP-9 in comparison with the respective control sample was observed. Based on the above results, we concluded that there was no inhibition of either the secretion or synthesis of the studied metalloproteinases from the cells. It can also be concluded that normal renal cells differ in their secretion of gelatinase outside the cell from malignant cells. The synthesis of MMP-2 may be at a similar level for both cell types, but malignant cells restrict the secretion of the enzyme MMP-2 to the extracellular space. It is well known that protein turnover is much slower outside the cell than inside [5]. Gelatinase, like all other matrix metalloproteinases, is an extracellular enzyme, so the reduction in enzyme secretion caused by cancer cells may explain the low MMP-2 content in tumor tissue. On the other hand, the MMP-9 content was at a higher level in cancer cells than in healthy cells, so it can be assumed that, under these circumstances, cancer cells did not restrict the secretion of MMP-9 into the extracellular space.

The presentation of the actual activity of gelatinases makes it possible to compare the activity of the two analyzed gelatinases in the studied samples. The results show that MMP-9 is about a thousand times more active in the control samples than MMP-2, while it is less active in its tumor samples. Given the constant process of extracellular matrix remodeling involving metalloproteinases, MMP-9 appears to be significantly involved in maintaining ECM homeostasis in the physiological conditions of the kidney. A significant increase in the activity of both enzymes was observed with the increased tumor stage. However, only MMP-2 from the G3 stage renal cell carcinoma achieved actual activity similar to the control samples. Such an increase in the actual activity of both studied enzymes may be further evidence explaining their important role in carcinogenic processes. 

In the present study, we examined the activity of MMP-2 and MMP-9 in both grades of tumor cells. No relationship was found between the expression/activity of the tested enzymes and tumor progression.

The results obtained from calculating the specific activity of each gelatinase indicate what part of it is in the active form, that is, without the inhibitor bound to its active site. Given both investigated gelatinases, MMP-9 shows much higher specific activity in the control samples than MMP-2. However, an extremely large decrease compared with the control samples in the specific activity of MMP-9 in both grades of kidney cancer was found. Significantly higher specific activity from MMP-2 in the cancer samples in comparison with MMP-9 was also identified, with its increase in a higher grade of cancer. Putting this all together, it appears that most MMP-2 molecules remain in an active form, unlike MMP-9. The enzymatic activity appears to be exerted to a greater extent by MMP-9 than by MMP-2 proteins.

Based on Western blot analysis, both tested gelatinases were found to be present primarily in high-molecular-weight complexes in both control samples and tumor samples of both stages. It was noted that these complexes break down under the influence of disulfide bridge-reducing agents, which means that the bonds connecting all the components of the mentioned complexes are rather weak and have a non-covalent nature.

MMP-2 in its free and active form was found to be present in all examined tissues, both the control samples and tumor samples, as a band with a molecular weight of about 60–65 kDa but occurring only after a reduction in disulfide bonds. The results indicate the presence of MMP-9 in a free and active form in all tested tissues, as determined by the presence of a very narrow and poorly visible band with a molecular weight of more than 80 kDa. Compared with results from other authors, we found an increase in the expression of tumor cell gelatinases in patients with renal cell carcinoma [16,17]. Kallakury et al. [18] indicated a correlation between the increased expression of both gelatinases, disease progression, and poor prognosis. In addition, a study by Amin et al. [19] noted a correlation between the increased expression of the tested enzymes and a higher grade in the TNM (Tumor Nodes Metastases) grading system. However, other authors have not noted a significant correlation between gelatinase expression and tumor malignancy grade [20,21,22]. 

Despite the high MMP-9 content in the G3 phase of kidney cancer, its activity was much lower than that of MMP-2 and the control samples. This may suggest that the catalytic ability of MMP-9 is much lower than MMP-2. Despite the much lower content, gelatinase A seems to be the dominant gelatinase in kidney cancer. On the other hand, the differences between the results obtained for the two tested gelatinases may suggest that these enzymes have their specific involvement in the strict period of tumor growth and its differentiation. Moreover, the above-mentioned results indicate that G3 renal tumor cells may enhance gelatinase activity. Based on the obtained results, MMP-9 showed higher content and lower specific activity compared with MMP-2. Therefore, it can be assumed that more MMP-9 molecules were present in the tested samples in the active form than in the inactive form. 

## 5. Limitations of the Study

The control tissue was taken from the same kidney, so the influence of the development of the tumor process on the metabolism of the whole kidney and, therefore, on the control fragment cannot be excluded. Moreover, a limited number of participants may not accurately represent the diversity and characteristics of the larger population. Regrettably, not all the collected patient tissues met the criteria for inclusion in a specific study group, which notably prolonged the duration of the sample collection process. However, it is essential to highlight that the sample size was sufficient to perform statistical analysis. Another limitation of our study was the challenge of conducting measurements of MMP-2 or MMP-9 activity. This difficulty arose because of the absence of a dedicated substrate for each of these MMPs. To address this issue, we employed a method where MMP-2 or MMP-9 from a test sample was bound to a specific antibody immobilized on a fluorimetric plate. Subsequently, the unbound MMPs from the sample were washed away. 

## 6. Conclusions

Understanding the role of gelatinases in ECM degradation is crucial for gaining insights into the pathomechanism of renal cancer development. Our study reveals significant findings that have implications for human renal cell carcinoma. Differences in the activity of the two gelatinases were shown. The results indicate their opposite action both at the stage of ECM remodeling and at different stages of renal tumor development. It can be assumed that the initiation of collagen degradation in the extracellular matrix is the most important step in the process of tumor growth. It should be emphasized that the measurement of MMP content and activity directly from kidney samples reflects changes occurring exactly in that specific tissue. Conversely, the changes in enzyme content and activity in body fluids reflect the state of the whole body, not a specific single tissue. In the future, it will be necessary to compare the results of tissue determinations with the results of serum and urine determinations in the same study group. This will help determine whether MMPs can be good diagnostic and prognostic biomarkers of kidney cancer. Moreover, further studies are necessary to confirm the potential therapeutic targeting of MMP-2 and MMP-9 owing to their involvement in tumor invasion tissue remodeling.

## Figures and Tables

**Table 1:** Clinicopathological characteristics of patients.

GRADE 2					GRADE 3				
Patient Number	Sex	Age (Years)	BMI	Tumor Size (cm)	Patient Number	Sex	Age (Years)	BMI	Tumor Size (cm)
1	F	73	23.5	5.0	1	M	78	26.5	12.0
2	F	64	24.8	5.0	2	M	51	28.9	3.5
3	M	48	24.1	3.5	3	M	62	31.6	7.0
4	M	70	27.7	4.0	4	M	53	29.9	4.0
5	M	68	26.6	5.5	5	M	77	25.3	9.5
6	M	63	24.2	5.5	6	F	82	24.9	5.0
7	F	75	25.9	4.5	7	M	62	31.3	8.0
8	M	53	29.6	5.5	8	M	72	28.6	6.5
9	F	67	23.1	8.5	9	M	73	24.2	10.5
10	M	74	27.8	6.0	10	F	53	28.9	5.5

F, female; M, male; BMI, Body Mass Index.

## Data Availability

The data that support the findings will be available on request under the corresponding author’s e-mail: mlynarz36@yahoo.pl.

## Supplementary Materials

The following supporting information can be downloaded at https://www.mdpi.com/article/10.3390/cancers15225475/s1. Table S1: The results of all measurements and statistical analysis.
